# Balloon Guide Catheter Use and Outcomes After Endovascular Thrombectomy for Ischemic Stroke Due to Large Vessel Occlusions

**DOI:** 10.1007/s00062-025-01570-z

**Published:** 2025-09-22

**Authors:** Björn M. Hansen, Emma Hall, Alex Szolics, Tommy Andersson, Johan Wassélius

**Affiliations:** 1 Department of Radiology, Skåne University Hospital, 22185 Lund Lund, Sweden; 2 Stroke Imaging Research Group, Department of Clinical Sciences Lund, Lund University, Lund, Sweden; 3 Department of Neuroradiology, Karolinska University Hospital, Solna, Sweden; 4 Department of Clinical Neuroscience, Karolinska Institutet, Stockholm, Sweden

**Keywords:** Stroke, Device, Reperfusion, Technique, Thrombectomy

## Abstract

**Background:**

Balloon-guide catheters (BGC) have been associated with improved procedural and functional outcomes following endovascular thrombectomy (EVT) in multiple observational studies. Recently the PROTECT-MT trial challenged this benefit, showing worse functional outcomes when using BGCs. This study aims to assess the association between BGC-use and procedural and functional outcomes in a large real-world cohort.

**Methods:**

Patients who underwent EVT for anterior circulation large vessel occlusion (LVO) between 2017 and 2021 were included in two Swedish registries: EVAS and Riksstroke. Outcomes included recanalization success (modified Treatment In Cerebral Infarction [mTICI] 2b/2c–3), good 90-day functional outcome (modified Rankin Scale score 0–2), and safety outcomes (iatrogenic dissection, perioperative embolization, early neurological deterioration, and 90-day mortality). Subgroup analysis was conducted by first-line EVT strategy: stent-retriever (with/without aspiration) or contact aspiration alone.

**Results:**

Of 4843 patients, 2483 met the inclusion criteria. BGCs were used in 1449 cases (58.4%) and were more frequently used with stent-retrievers (83.0%) than with contact aspiration (24.1%, *p* < 0.001). BGC-use was associated with higher rates of excellent recanalization (mTICI 2c–3) and first-pass success, particularly in stent-retriever cases (*p* < 0.001), but not with good recanalization (mTICI 2b–3). No association was found with good 90-day functional outcome or any safety outcome (*p* > 0.05). A numerically lower mortality rate was observed with BGCs (19.0% vs. 22.9%, *p* = 0.019), although this was not significant after adjustment.

**Conclusions:**

BGC-use during EVT was associated with excellent recanalization and first-pass reperfusion, primarily in stent-retriever-treated patients. No significant impact on 90-day functional outcome nor on safety indicators was observed.

**Supplementary Information:**

The online version of this article (10.1007/s00062-025-01570-z) contains supplementary material, which is available to authorized users.

## Introduction

Temporary flow-arrest and/or flow-reversal using a balloon-guide catheter (BGC) may increase success-rate and prevent distal embolization during endovascular thrombectomy (EVT), procedural benefits that were initially shown *in-vitro* [1, 2]. The benefit of BGCs for procedural and functional outcomes in anterior circulation ischemic stroke due to large vessel occlusion (LVO) has been shown in multiple observational studies [3–5] and in several meta-analyses [6, 7].

In contrast, the recent randomized controlled trial (RCT) PROTECT-MT (Proximal Temporary Occlusion using Balloon Guide Catheter for Mechanical Thrombectomy), conducted in China and including 329 patients, was terminated early as the use of BGCs was associated with worse functional outcomes and showed a trend towards increased all-cause mortality in interim analysis [8]. In another RCT ProFATE (Proximal Blood Flow Arrest During Endovascular Thrombectomy), including 134 patients from the UK, BGC use did not improve the frequency of excellent recanalization (extended Thrombolysis in Cerebral Infarction score [TICI] 2c–3). However, in secondary outcome analyses flow arrest was associated with a lower rate of distal embolization, higher rate of complete recanalization (extended TICI 3) at the first attempt, lower 90-day mortality rates and a trend towards lower incidence of symptomatic hemorrhage, while no safety issues were observed [9].

In view of the conflicting results between these recent and previous observational studies, we aimed to assess the association between the use of BGC and procedural and functional outcomes in a large real-world consecutive population with universal access to health care.

## Methods

### Study Design

We performed a nationwide, register-based observational study utilizing data between 2017 and 2021 from the Swedish EndoVAscular treatment of Acute Stroke (EVAS) registry and the national quality registry for stroke care, Riksstroke.

### Data Sources

EVAS prospectively collects clinical and procedural data from all seven comprehensive stroke centers in Sweden, with a coverage rate of > 98.5% of all EVT procedures performed during the study period [10]. Riksstroke collects clinical and outcome data from all 72 Swedish centers providing acute stroke care with consistent coverage of > 90% of all in-hospital admissions for acute stroke [11].

Stroke characteristics data, including occlusion location, procedural and treatment details, complications, pre- and post-EVT stroke severity, were obtained from EVAS. Functional 90-days outcome, including mortality, was collected from Rikstroke. [REMOVED] approved the study and waived informed consent.

### Patient Selection

Patients treated with EVT for an anterior circulation large vessel occlusion (LVO) in the distal internal carotid artery (I- or T‑occlusions) or the M1-segment of the middle cerebral artery (MCA) were included in the study. If a patient had more than one EVT within three months, only the first was included.

### Variables

The degree of final revascularization was categorized as *good* (modified TICI [mTICI] 2b–3) or *excellent* (mTICI 2c–3) based on the final digital subtraction angiographic run [12]. First-pass reperfusion (FPR) was defined as mTICI 2c–3 after the first treatment attempt. Analyses on excellent revascularization and FPR were restricted to patients treated in 2018–2021 as the 2c-grade was only available from 2018 onwards.

Patients where direct contact aspiration or stent-retriever with- or without distal aspiration was used as the primary strategy were included.

Iatrogenic internal carotid artery dissection and embolization to new vascular territory were reported as perioperative complications. Early neurological deterioration was defined as a worsening on the National Institutes of Health Stroke Scale (NIHSS) by ≥ 4 points within 24 h of the EVT as compared to baseline.

The extent of infarction at baseline-CT was categorized into the four modified ASPECTS (mASPECTS) groups: 10 (no ischemic injury); 7–9 (infarct in < 1/3 of the MCA territory *or* in the basal ganglia); 5–6 (infarct in < 1/3 of the MCA territory *and* in the basal ganglia); 0–4 (infarct in > 1/3 of the MCA territory), based on the available infarct classification in the EVAS-registry [13].

Functional outcome at 90-days in Riksstroke is collected by a standardized follow-up questionnaire which is converted into the modified Rankin Scale (mRS) using a validated algorithm [14, 15]. Good functional outcome was classified as mRS 0–2. Riksstroke also incorporates official census data from the National Cause of Death Register, which was used to assess 90-day mortality.

### Statistical Analysis

The association between BGC-use and baseline characteristics and peri- and postprocedural outcomes were analyzed. Descriptive analyses were analyzed using Pearson’s χ2 test and Mann-Whitney U test, where appropriate. Two-sided *p*-values < 0.05 were regarded as statistically significant. Logistic regression analyses were used for uni- and multivariate analyses, adjusting for the prespecified factors age, sex, baseline NIHSS-score, and baseline mASPECTS. Subgroup analyses were conducted based on first line strategy: contact aspiration or stent-retriever (with- or without distal aspiration), and internal carotid artery or M1-segment occlusion location.

Analyses on functional outcomes were restricted to pre-stroke functionally independent patients (mRS 0–2). Multiple imputations by chained equations with age, sex, pre-stroke mRS, baseline NIHSS-score, mASPECT, NIHSS-score 24 h after EVT, and degree of revascularization, as predictive values were used to account for missing data on 90-day functional outcome for patients that were known to be alive at that time-point. Twenty complete datasets were constructed and the estimates from each imputation were combined using Rubin’s rule. The primary outcome analysis was also performed on the original dataset prior to multiple imputations.

## Results

**Fig. 1:**
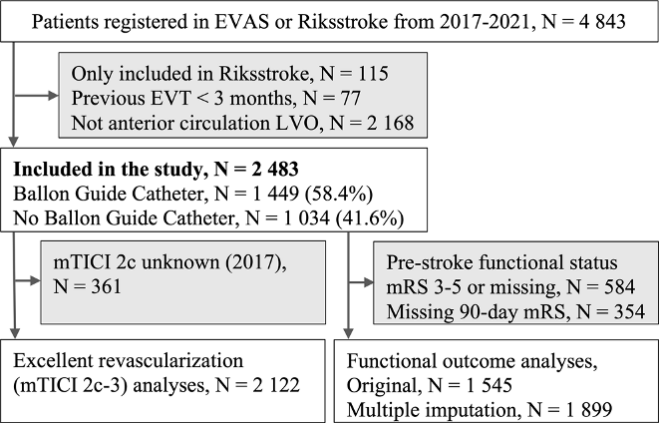
Consort diagram illustrating study patient selection. Abbreviations: *EVT* Endovascular thrombectomy, *LVO* Large Vessel Occlusion, *mTICI* modified Thrombolysis in Cerebral Infarction scale, *mRS* modified Ranking Scale

Of all 4843 patients included in EVAS during 2017–2021, 2483 (51.3%) underwent EVT for anterior circulation LVOs, with BGCs used in 1449 cases (58.4%) (Fig. 1). The number of included patients per center ranged from 20 to 666 and the corresponding percentage of BGC usage varied between 0 and 92% (Supplemental Table S‑1). The most used BGCs were the 8F Flowgate (811/1449, 56.0%), the 9F Merci (662/1449, 45.7%) and the 9F Cello (106/1449, 7.3%).

Patients in the BGC group were younger and had longer onset-to-puncture times (Table 1). Additionally, T‑occlusions, intravenous thrombolysis, and the use of conscious sedation, were more common in the BGC-group. The procedure-related times were similar between the groups. BGCs were more frequently used in combination with stent-retrievers as first-line strategy (1164/1403, 83.0%) as compared with contact aspiration (209/866, 24.1%, *p* < 0.001).

**Table 1:** Baseline characteristics and procedure related factors among patients treated endovascular thrombectomy with or without a ballon guide catheter (BGC).

Variable	BGC-use (*N* = 1449)	Non-BGC (*N* = 1034)	Total (*N* = 2483)	Missing *N* (%)	*p*-value
Age, years (IQR)	74 (65–82)	76 (67–83)	75 (66–83)	62 (2.5%)	0.004
Female (%)	745 (51.4%)	524 (50.7%)	1269 (51.1%)	5 (0.2%)	0.745
Baseline NIHSS score (IQR)	17 (12–20)	17 (12–21)	17 (12–20)	56 (2.3%)	0.499
mASPECTS 7–10 (%)	1215 (83.8%)	841 (81.3%)	2056 (82.3%)	133 (5.4%)	0.055
Intravenous thrombolysis (%)	629 (43.4%)	415 (40.1%)	1044 (42.0%)	30 (1.2%)	0.040
Extracranial ICA occluded (%)	138 (9.5%)	118 (11.4%)	256 (10.3%)	0 (0.0%)	0.127
*Occlusion location*				0 (0.0%)	–
I‑occlusion	129 (8.9%)	119 (11.5%)	248 (10.0%)	–	0.033
T‑occlusion	266 (18.4%)	131 (12.7%)	397 (16.0%)	–	< 0.001
M1-occlusion	1054 (72.7%)	784 (75.8%)	1838 (74.0%)	–	0.086
*Stroke onset to puncture*				321 (12.9%)	–
Time (IQR) hh:mm	04:03 (02:40–06:32)	03:45 (02:27–06:04)	03:55 (02:35–06:25)	–	0.035
Within 6 h (%)	905 (62.5%)	656 (63.4%)	1561 (62.9%)	–	0.052
*Puncture to revascularization*				13 (0.5%)	–
Time (IQR) hh:mm	00:45 (00.26–01:18)	00:43 (00:23–01:17)	00:44 (00:25–01:18)	–	0.256
*Anesthesia form (%)*				4 (0.2%)	–
General anesthesia (GA)	444 (30.6%)	510 (49.3%)	954 (38.4%)	–	< 0.001
Conscious sedation (CS)	979 (67.6%)	484 (46.8%)	1463 (58.9%)	–	< 0.001
Converted from CS to GA	25 (1.7%)	37 (3.6%)	62 (2.5%)	–	0.004
*First-line strategy (%)*				214 (8.6%)	–
Contact aspiration only	209 (14.4%)	657 (63.5%)	866 (34.9%)	–	< 0.001
Stent-retriever with or without contact aspiration	1164 (80.3%)	239 (23.1%)	1403 (56.5%)	–	< 0.001

*BGC* Balloon Guide Catheter, *IQR* Inter Quartal Range, *NIHSS* National Institutes of Health Stroke Scale, *mASPECTS* modified ASPECT score, *ICA* Internal Carotid Artery

### Revascularization and First-pass Recanalization

Excellent recanalization (mTICI 2c–3) was more common among patients where BGCs were used (BGC 68.5% vs. non-BGC 63.4%, *p* = 0.016), as shown in Table 2. BGC use was associated with improved success-rate in procedures with stent-retriever as first-line strategy (BGC 67.8% vs. non-BGC 52.0%, *p* < 0.001), but not for procedures with contact aspiration as first-line strategy (BGC 70.5% vs. non-BGC 71.0%, *p* = 0.979), see Supplemental Tables S‑2 and S‑3 for further details. There were similar trends for patients treated for a M1-segment occlusion (BGC 68.8% vs non-BGC 64.0%, *p* = 0.043) and for ICA occlusions (BGC 66.8% vs non-BGC 61.3%, *p* = 0.167), see Supplemental Tables S‑4 and S‑5.

**Table 2:** Peri- and postprocedural outcomes in patients treated with endovascular thrombectomy with or without a ballon guide catheter (BGC).

Variable	BGC-use (*N* = 1449)	Non-BGC (*N* = 1034)	Total (*N* = 2483)	*p*-value
Iatrogenic ICA dissection	18 (1.2%)	10 (1.0%)	28 (1.1%)	0.522
Embolization to new territory	71 (4.9%)	38 (3.7%)	109 (4.4)	0.142
Good revascularization (mTICI 2b–3)	1267 (87.4%)	887 (85.8%)	2154 (86.7%)	0.266
Excellent recanalization (mTICI 2c–3)*	851 (68.5%)	555 (63.4%)	1406 (66.3%)	0.016
First-pass reperfusion (mTICI 2c–3)*	499 (40.0%)	312 (35.6%)	811 (38.2%)	0.039
Early neurological deterioration	94 (6.5%)	67 (6.5%)	161 (6.5%)	0.935
Good 90-day functional outcome, MI**	464 (40.3%)	303 (40.5%)	767 (40.3%)	0.947
Good 90-day functional outcome, original dataset**	372 (40.0%)	240 (39.0%)	612 (39.6%)	0.670
Death at 90-day follow-up	276 (19.0%)	237 (22.9%)	513 (20.7%)	0.019

*BGC* Balloon Guide Catheter, *ICA* Internal Carotid Artery, *IQR* Inter Quartal Range, *mTICI* modified Thrombolysis in Cerebral Infarction score, *MI* Multiple Imputations

*Available for 2018–2021, *n* = 2122

**Patients with known pre-stroke mRS 0–2 and 90-day mRS follow-up in the original dataset (*n* = 1 545) and after MI (*n* = 1899)

Similarly, BGC use was associated with a higher rate of first-pass recanalization (40.0% compared to 35.6%, *p* = 0.039). When analyzing first-pass recanalization based on first-line strategy, a large benefit was seen in procedures with stent-retriever as the first-line strategy (BGC 40.4% vs. non-BGC 25.0%, *p* < 0.001), but there was no statistically significant difference in the contact aspiration-group (BGC 50.5% vs. non-BGC 46.3%, *p* = 0.320). While there was an association between first-pass recanalization and BGC use among patients with ICA occlusions (BGC 35.7% vs non-BGC 21.2%, *p* < 0.001), no difference was observed for patients with M1-occlusions (BGC 41.7% vs 40.0%, *p* = 0.510).

No statistically significant difference in the frequency of good revascularization (mTICI 2b–3) was observed between the groups (BGC 87.4% vs. non-BGC 85.8%, *p* = 0.266).

### Safety Outcomes

The rates of iatrogenic ICA dissections were similar between the BGC and non-BGC groups at 1.2 and 1.0%, respectively (*p* = 0.522). The difference in rates of perioperative embolization to a new vascular territory was small and not statistically significant (BGC 4.9% vs. non-BGC: 3.7%, *p* = 0.142), as shown in Table 2. The rates of early neurological deterioration were 6.5% in both groups.

### Functional Outcome and Mortality at 90 Days

Of the total study population, 1899 (76.5%) patients were functionally independent (mRS 0–2) before their stroke and were included in the functional outcome analysis. Of these, 354 (18.6%) were confirmed to be alive at 90 days, but their 90-day functional status was missing and was therefore imputed.

The rates of good functional outcome (mRS 0–2) were similar between groups, both in the original dataset (BGC: 40.0% vs. non-BGC: 39.0%) and when multiple imputation was used to account for missing data (BGC: 40.3% vs. non-BGC: 40.5%). The use of BGC was not associated with increased or reduced odds for good 90-day functional outcome in the original dataset (OR 1.05, CI 95% 0.85–1.29) nor after multiple imputation (OR 0.99, CI 95% 0.81–1.21).

These results remained largely unchanged in multivariate analyses of the original dataset (OR 0.90, CI 95% 0.71–1.13), as well as after multiple imputations (OR 0.96, CI 95% 0.78–1.19) and when analyses were stratified on first-line strategy and occlusion location (data not shown).

The 90-day mortality-rate was numerically lower in the BGC-group 19.0% vs non-BGC-group 22.9% (*p* = 0.019). The lower odds for death at 90-days in the BGC-group (OR: 0.79, CI 95%: 0.65–0.96; *p* = 0.019) did, however, not remain after adjusting for preselected potential confounders in the multivariate analysis (OR: 0.86, CI 95% 0.69–1.073).

## Discussion

In this study we analyze the potential procedural and outcome benefits of BGC use in EVT for anterior circulation LVOs in routine healthcare, based on a real-world registry with high national coverage (> 98.5%). BGC use was associated with improved recanalization and first-pass reperfusion in stent-retriever-treated patients. However, no significant impact on 90-day functional outcome was observed.

The main rationale for this study was the recently reported results from the PROTECT-MT trial that was terminated early due to safety concerns suggesting that BGC use may be associated with worse functional outcomes and numerically increased mortality [8], contrary to previous observational studies that has unanimously shown a benefit of BGC use in EVT for anterior circulation LVOs [3–7]. In contrast to the PROTECT-MT trial, we did not observe that BGC use led to longer puncture to reperfusion times, nor increased mortality or decreased functional outcomes [8].

Our result reflects EVT use in a real-world population and show a benefit from BGC-use for excellent recanalization (mTICI ≥ 2c) and first-pass recanalization, which is in-line with the positive trends for the flow-arrest group in the ProFATE trial [9]. However, the benefit in our study was mainly in procedures where stent-retrievers, with or with-out contact aspiration catheters were used as first-line strategy, whereas no procedural benefit was seen when contact aspiration only was used as first-line strategy. Similarly, the positive association between first-pass recanalization and BGC use in our study was primarily driven by the smaller subgroup of patients with carotid occlusions, whereas no such association was observed in patients with M1 occlusions.

The benefit in excellent recanalization in our study is in line with previous *in-vitro* findings, showing that flow arrest/reversal by BGCs reduces the number of embolic fragments that are shaved off and embolized when the clot is engulfed into the guide catheter [2]. However, stent-retrievers were primarily used without contact aspiration in the *in-vitro* studies and in the early clinical studies, whereas today stent-retrievers are often used in combination with both aspiration catheters and BGCs, and it is possible that the use of contact aspiration reduces the benefit of BGCs [16] despite that one previous study suggested that the effect persists [17].

The degree of recanalization has been shown to be the most important predictor of good clinical outcome [18] and failed recanalization also is proven to increase complications, including symptomatic intracranial hemorrhage (sICH) [13]. Furthermore, the functional outcome benefit of improving final recanalization from good (mTICI ≥ 2b) to excellent (mTICI ≥ 2c) is well-established [18], and increased excellent recanalization rates and first-pass recanalization rates are therefore desirable.

However, in our study the 5.1% increased rate of excellent recanalization in the BGC-group did not translate into significantly improved functional outcomes which may indicate that either the potential effect size is small, or that the harm of flow-arrest may outweigh the benefit in patients with well-developed arterial collaterals. Finally, the mRS may be too crude and consequently unable to capture more subtle neurological impairments associated with microscopic emboli affecting the peripheral circulation.

### Limitations

This study has several limitations. The mTICI is self-reported by the operator or the performing center and not core-lab adjudicated which may lead to and over- or underreporting of the degree of revascularization, which may introduce a systematic error. Contact aspiration alone as the first attempt is registered in EVAS, but it is not possible to distinguish between stent-retriever only and combined techniques (stent-retriever and contact aspiration), therefore, the two are grouped. The use of BGCs were based on operator choice with respect to anatomical features and personal preferences, as-well-as local routines at each center, which may have introduced a selection bias.

The EVAS-registry currently does not register EVT strategy as an independent variable, and consequently the strategy for the first attempt was constructed by several other variables, and it was not possible to analyze different EVT strategies beyond the initial choice of strategy.

## Conclusions

This study confirms earlier observational studies showing a significant association between BGC use and excellent recanalization- and first-pass recanalization rates. In contrast the recent findings in the PROTECT-MT trial no worrying safety signals were observed. The benefit of BGC use was generally propelled by procedures were stent-retriever, with or without contact aspiration, were used as first-line strategy. A future registry-based RCT [19] may be able to provide sufficient sample-size to determine the real-world effectiveness of BGC use in EVT.

## Supplementary Information

Supplemental Tables 1–5
