# Supplementary material for: Balloon Guide Catheter Use and Outcomes After Endovascular Thrombectomy for Ischemic Stroke Due to Large Vessel Occlusions
Source: Clin Neuroradiol. 2025 Sep 22;36(1):177–83. doi: 10.1007/s00062-025-01570-z (PMC13009045; doi:10.1007/s00062-025-01570-z)
Supplement: Supplementary file 1 — Supplemental Tables 1–5 [file 62_2025_1570_MOESM1_ESM.docx]

**SUPPLEMENTAL MATERIAL**

**Title: Balloon Guide Catheter Use and Outcomes After Endovascular Thrombectomy for Ischemic Stroke due to Large Vessel Occlusions**

**Supplemental Table 1 – Inclusion by Participating Centers.** Summary of the number of patients treated with endovascular thrombectomy with or without a ballon guide catheter (BGC) per center (2017-2021).

| **Variable** | **BGC**  (N=1449) | **No BGC**  (N=1034) | **Total**  (N=2483) |
| --- | --- | --- | --- |
| Karolinska University Hospital | 524 (81.0%) | 123 (19.0%) | 647 |
| Linköping University Hospital | 12 (9.8%) | 110 (90.2%) | 122 |
| Sahlgrenska University Hospital | 6 (0.9%) | 660 (99.1%) | 666 |
| Skåne University Hospital | 562 (91.7%) | 51 (8.3%) | 613 |
| University Hospital of Umeå | 124 (80.0%) | 31 (20.0%) | 155 |
| Uppsala University Hospital | 221 (85.0%) | 39 (15.0%) | 260 |
| Örebro University Hospital | 0 (0.0%) | 20 (100%) | 20 |

**Supplemental Table 2 – Stent-retriever with or without contact aspiration as first-line strategy.** Peri- and postprocedural outcomes in patients treated with endovascular thrombectomy, using stent-retriever as first strategy, with or without a ballon guide catheter (BGC).

| **Variable** | **BGC-use**  (N=1164) | **Non-BGC**  (N=239) | **Total**  (N=1403) | **p-value** |
| --- | --- | --- | --- | --- |
| Dissection extradural ICA | 16 (1.4%) | 2 (0.8%) | 18 (1.3%) | 0.501 |
| Embolization to new territory | 53 (4.6%) | 13 (5.4%) | 66 (4.7%) | 0.556 |
| Good revascularization  (mTICI 2b-3) | 1015 (87.1%) | 208 (87.0%) | 1223 (87.2%) | 0.886 |
| Excellent recanalization  (mTICI 2c-3)* | 675 (67.8%) | 106 (52.0%) | 781 (65.1%) | <0.001 |
| First-pass reperfusion  (mTICI 2c-3)* | 402 (40.4%) | 51 (25.0%) | 453 (37.7%) | <0.001 |
| Early neurological deterioration | 73 (7.2%) | 22 (10.6%) | 95 (7.8%) | 0.097 |
| Good 90-day functional outcome, MI** | 374 (40.9%) | 79 (42.9%) | 453 (41.2%) | 0.629 |
| Good 90-day functional outcome, original dataset** | 299 (40.6%) | 63 (41.4%) | 362 (40.7%) | 0.841 |
| Death at 90-day follow-up | 232 (19.9%) | 55 (23.0%) | 287 (20.4%) | 0.282 |
| *Available for 2018-2021, n=1201 (BGC n=997; non-BGC n=204); ** Patients with known pre-stroke mRS 0-2 and 90-day mRS follow-up in the original dataset (n=889) and after MI (n=1100) | | | | |

**Supplemental table 3 – Contact aspiration only as first-line strategy.** Peri- and postprocedural outcomes in patients treated with endovascular thrombectomy, using contact-aspiration as first strategy, with or without a ballon guide catheter (BGC).

| **Variable** | **BGC-use** (N=209) | **Non-BGC** (N=657) | **Total**  (N=866) | **p-value** |
| --- | --- | --- | --- | --- |
| Dissection extradural ICA | 2 (1.0%) | 7 (1.1%) | 9 (1.0%) | 0.893 |
| Embolization to new territory | 14 (6.7%) | 20 (3.0%) | 34 (3.9%) | 0.018 |
| Good revascularization (mTICI 2b-3) | 183 (87.6%) | 603 (91.8%) | 786 (90.8%) | 0.173 |
| Excellent recanalization (mTICI 2c-3)* | 134 (70.5%) | 400 (71.0%) | 534 (70.9%) | 0.979 |
| First-pass reperfusion  (mTICI 2c-3)* | 96 (50.5%) | 261 (46.3%) | 357 (47.4%) | 0.320 |
| Early neurological deterioration | 16 (7.7%) | 30 (4.6%) | 46 (5.3%) | 0.061 |
| Good 90-day functional outcome, MI** | 69 (39.4%) | 186 (40.0%) | 255 (39.8%) | 0.866 |
| Good 90-day functional outcome, original dataset** | 56 (39.2%) | 147 (38.7%) | 203 (38.8%) | 0.921 |
| Death at 90-day follow-up | 33 (15.8%) | 149 (22.7%) | 182 (21.0%) | 0.033 |
| *Available for 2018-2021, n=753 (BGC n=190; non-BGC n=563); ** Patients with known pre-stroke mRS 0-2 and 90-day mRS follow-up in the original dataset (n=523) and after MI (n=640) | | | | |

**Supplemental table 4 – M1-segment Middle Cerebral Artery Occlusions.** Peri- and postprocedural outcomes in patients treated with endovascular thrombectomy for M1-segment occlusions, with or without a ballon guide catheter (BGC).

| **Variable** | **BGC-use** (N=1054) | **Non-BGC** (N=784) | **Total**  (N=1838) | **p-value** |
| --- | --- | --- | --- | --- |
| Dissection extradural ICA | 11 (1.0%) | 8 (1.0%) | 19 (1.0%) | 0.961 |
| Embolization to new territory | 41 (3.9%) | 23 (2.9%) | 64 (3.5%) | 0.269 |
| Good revascularization (mTICI 2b-3) | 923 (87.6%) | 681(86.9%) | 1604 (87.3%) | 0.880 |
| Excellent recanalization (mTICI 2c-3)* | 619 (68.8%) | 428 (64.0%) | 1047 (66.8%) | 0.043 |
| First-pass reperfusion (mTICI 2c-3)* | 375 (41.7%) | 268 (40.0%) | 643 (41.0%) | 0.510 |
| Early neurological deterioration | 60 (5.7%) | 48 (6.1%) | 108 (5.9%) | 0.718 |
| Good 90-day functional outcome, MI** | 363 (43.7%) | 243 (42.6%) | 606 (43.3%) | 0.716 |
| Good 90-day functional outcome, original dataset** | 293 (43.6%) | 194 (41.3%) | 487 (42.7%) | 0.422 |
| Death at 90-day follow-up | 192 (18.2%) | 161 (20.5%) | 353 (19.2%) | 0.212 |
| *Available for 2018-2021, n=1568 (BGC n=899; non-BGC n=669); ** Patients with known pre-stroke mRS 0-2 and 90-day mRS follow-up in the original dataset (n=1141) and after MI (n=1400) | | | | |

**Supplemental table 5 – Internal Cerebral Artery Occlusions.** Peri- and postprocedural outcomes in patients treated with endovascular thrombectomy for Internal Cerebral Artery (ICA-I and ICA-T) occlusions, with or without a ballon guide catheter (BGC).

| **Variable** | **BGC-use** (N=395) | **Non-BGC** (N=250) | **Total**  (N=645) | **p-value** |
| --- | --- | --- | --- | --- |
| Dissection extradural ICA | 7 (1.7%) | 2 (0.8%) | 9 (1.4%) | 0.305 |
| Embolization to new territory | 30 (7.6%) | 15 (6.0%) | 45 (7.0%) | 0.439 |
| Good revascularization (mTICI 2b-3) | 344 (87.1%) | 206 (82.4%) | 550 (85.3%) | 0.051 |
| Excellent recanalization (mTICI 2c-3)* | 232 (66.8%) | 127 (61.3%) | 359 (64.8%) | 0.167 |
| First-pass reperfusion (mTICI 2c-3)* | 124 (35.7%) | 44 (21.2%) | 168 (30.3%) | <0.001 |
| Early neurological deterioration | 34 (8.6%) | 19 (7.6%) | 53 (8.2%) | 0.872 |
| Good 90-day functional outcome, MI** | 101 (31.5%) | 60 (33.7%) | 161 1(32.3%) | 0.672 |
| Good 90-day functional outcome, original dataset** | 79 (30.6%) | 46 (31.5%) | 125 (30.9%) | 0.853 |
| Death at 90-day follow-up | 84 (21.3%) | 76 (30.4%) | 160 (24.8%) | 0.009 |
| *Available for 2018-2021, n=554 (BGC n=347; non-BGC n=207); ** Patients with known pre-stroke mRS 0-2 and 90-day mRS follow-up in the original dataset (n=404) and after MI (n=499) | | | | |
